# In vivo assessment of subcutaneous fat in dogs by real-time ultrasonography and image analysis

**DOI:** 10.1186/s13028-016-0239-y

**Published:** 2016-10-20

**Authors:** Rita Payan-Carreira, Luis Martins, Sónia Miranda, Pedro Olivério, Severiano R. Silva

**Affiliations:** 1 Zootecnia Department, CECAV-Centro de Ciência Animal e Veterinária, Universidade de Trás-os-Montes e Alto Douro, Quinta de Prados, 5000-801 Vila Real, Portugal; 2 EUVG-Escola Universitária Vasco da Gama, Campus Universitário, Bloco B, Lordemão, 3020-210 Coimbra, Portugal; 3 HVBV-Hospital Veterinário do Baixo Vouga, Segadães, 3750-742 Águeda, Portugal

**Keywords:** Dog, Body condition score, Ultrasound, Subcutaneous fat thickness

## Abstract

**Background:**

Systems for estimating body condition score (BCS) are currently used in canine practice to monitor fatness levels. These tools are cheap and easy to use but lack the necessary precision to monitor small changes in body fat, particularly during weight control treatments or in research. The present work aims to study the application of real-time ultrasonography (RTU) together with image analysis in the assessment of subcutaneous fat depots in dogs. Ultrasound images were collected from five anatomical locations (chest, flank, abdomen, thigh and lumbar) from 28 healthy dogs of different breeds and with a body weight (BW) ranging from 5.2 to 33.0 kg. BCS was collected by visual appraisal using a 5-point scale. Subcutaneous fat thickness (SFT) was estimated from RTU images, using the average of three measurements taken in fat deposits located above the muscles represented in each image. Correlations were established between SFT and BW or BCS as well as a classification of BCS-based fatness [*overweight* (BCS = 4), *ideal* (BCS = 3) and *lean* (BCS = 2)].

**Results:**

SFT was found to differ between the five regions considered (P < 0.001). Abdomen and thigh were the areas displaying the widest variation for the different dogs included in the study and also those correlating most with BW, in contrast to the chest, which showed the least variation. Overall, a strong correlation was found between BCS and SFT. The highest correlations were established for the flank, abdomen and lumbar areas. In every anatomical area, a decrease in SFT was observed across all three BCS classes, ranging from 48 to 65 % among *overweight* and *ideal* dogs, and from 46 to 83 % among *ideal* and *lean* dogs.

**Conclusions:**

Preliminary data showed that within this population there was a strong correlation between BCS and SFT estimated from RTU images. It was also observed that RTU measurements for fat thickness differed among the anatomical points surveyed suggesting differences in their sensitivity to a change in BCS. The images displaying the best prediction value for fatness variations were those collected at the lumbar and abdomen areas.

## Background

Obesity is the most common nutritional disorder in dogs and is becoming a common health and welfare problem worldwide [1, 2]; it results from the intake of excessive dietary energy combined with poor lifestyle habits, particularly inactivity. According to different surveys, the prevalence of dog obesity ranges from 22 to 44 % [3]. In dogs, obesity is linked to both a shortened lifespan [4] and increased incidence of secondary diseases, including metabolic diseases, respiratory distress, hypertension, cardiac disease, neoplasia as well as orthopaedic and skin diseases [3, 5].

Surveying a dog’s condition is a key aspect of routine clinical examination. Estimating body condition score (BCS) is the most commonly used method to estimate fatness in dogs [6] and to monitor the response to weight loss programs in daily practice. Knowledge of body composition, in particular the percentage of body fat, provides useful information about the physical and metabolic status of animals [7], allowing proper advice to be given on feeding and weight reduction programs [2, 8].

Over the years, several methods have been developed to accurately measure and estimate the percentage of body fat, and also to facilitate understanding of the causes and effects of obesity. These include dual-energy X-ray absorptiometry [7, 9], bioelectrical impedance [9], computed tomography [10] and magnetic resonance imaging [11]. Nevertheless, implementing such methods in clinical practice is difficult or expensive. Practitioners need simple, semi-quantitative methods to support their work, which may explain why body condition scoring remains the tool mostly used on a routine daily basis. While this procedure is cheap, easy to use and reproducible among operators [6], it is a subjective technique and lacks sharp sensitivity, being unable to detect minor variations in body composition over time and correlating poorly with body weight [6].

Several BCS charts for dogs are available, which use 5-, 7- or 9-point scores to estimate the degree of fatness. Charts which give a higher score correlate better to the amount of body fat percentage obtained from more accurate methods [2]. Although the accuracy of the 5-point scale may be increased by adding half-scores between the whole scores, the fact is that in Portugal, as in Japan [2], most veterinarians use the whole score 5-point scale in routine daily practice. However, BCS’s sensitivity regarding a dog’s overall fatness is generally considered lower than more expensive methods, mainly due to the particular pattern of fat distribution in this species [2]. Notably, it is hard to quantify fatness levels in borderline cases between two consecutive scores.

A growing number of clinics now use alternative and more precise methods to assess body fat distribution in animals, such as computer tomography or magnetic resonance imaging [7, 10, 11]. However, such techniques are generally not applied to BCS since the initial price, operating costs and the equipment’s lack of mobility severely limit its use on a routine daily basis.

Over the last two decades, real-time ultrasonography (RTU) has become an increasingly important tool for the measurement of body fatness. RTU is used today in several domestic species to predict in vivo body fat covering [12–14]. It is widespread both in animal science and in clinical research, because of its relatively low cost, portability, robustness and easiness of use as well as its ability to obtain precise and highly reproducible images; moreover, it is well tolerated by animals and is well accepted by the public. Nevertheless, little information is available on the use of RTU to assess body fatness in dogs [15]. As the 5-point BCS system is not dependent on animals’ weight and essentially displays the thickness of their fatness covering [15], we hypothesised that SFT estimated through RTU images would reflect the level of fatness covering, and would thus also be relatively independent of the size of the dog.

Therefore, the present study intends to assess body fat depots from five different anatomical sites in dogs using RTU and image analysis, seeking to validate RTU as a predictor of body fatness by establishing a relationship between BCS and subcutaneous fat measurements obtained by RTU.

## Methods

### Animals

Data were collected from 28 privately-owned mature dogs randomly recruited according to their BCS from among the patients at a private veterinary hospital. All the animals were indoor pet dogs or guide dogs for the blind that were assessed on routine veterinary visits, for treatments such as neutering, vaccination or deworming. All the animals were submitted to a physical examination and considered free of underlying pathologies. A positive pregnancy diagnosis was the sole additional excluding criterion imposed for the study.

### Body size, weight and body condition score

The animals in the study were representative of both genders (14 males and 14 females) and also of different sizes (miniature-4; small-10; medium-14), weight (5.2–33.0 kg) and BCS (2–4, on a 5-point scale).

Two trained operators independently assigned the dogs’ BCS, using visual appraisal and palpation, according to Hill’s 5-point scale [16]. In the present study, BCS in dogs ranged from 2 (*lean* or *underweight*) to 4 (*overweight*). Additionally, dogs weight was assessed using an electronic scale displaying a sensitivity of 1 g.

Three morphometric variables were used to estimate the size of the dog, namely, height at withers, thoracic girth and the rump width. These were measured with the animal in standing position, looking straight ahead with the head in normal carriage position, using a measuring stick, callipers or a tape as appropriate. In the case of crossbred dogs, the height at withers and thoracic girth were used to establish the size of the dogs, following similarities with the body frame of breeds recognised by FCI (Fédération Cynologique Internationale) whose standards were used to define purebred size. In short, the height at withers and thoracic girth were used to distinguish between miniature and small sized dogs, while the height at withers was used to distinguish between small and medium sized dogs.

### Ultrasound image acquisition

To obtain RTU images, a General Electric ultrasound scanner (GE logic book XP, General Electrics, Buckinghamshire, UK) was used which was equipped with a 39 mm-long multifrequency linear transducer (8LRS, 6–11 MHz; General Electrics, Buckinghamshire, UK), set to 10 MHz. RTU image acquisition was performed in right lateral recumbency, without the need for sedation or anaesthesia. There was no need for hair clipping at the image collection spots; ethanol and ultrasound gel served as a coupling medium.

**Fig. 1:**
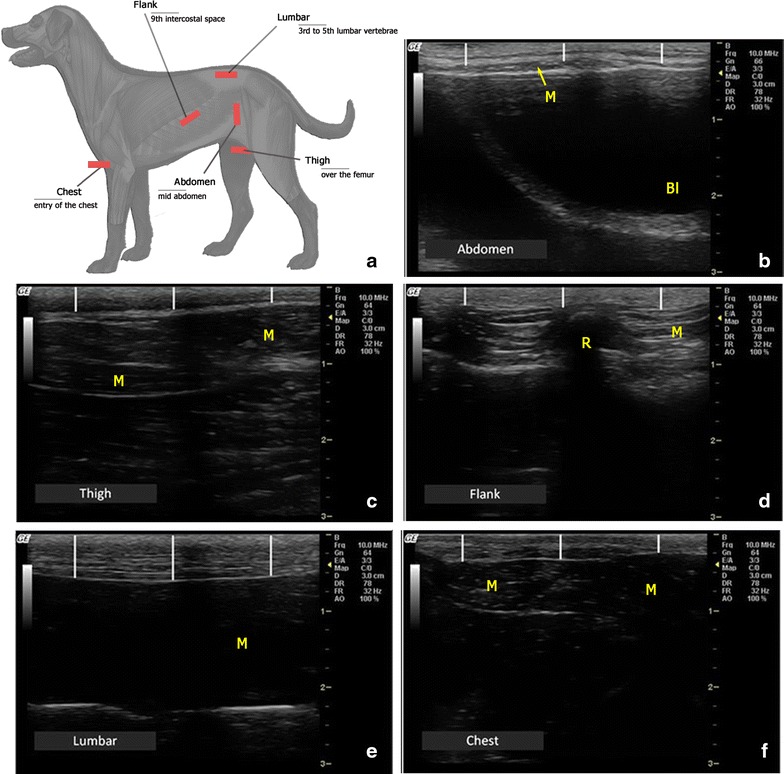
Real-time ultrasonography (RTU) assessment of subcutaneous fat thickness (SFT) in dogs. **a** Schematic representation of the anatomical sites used in the study to assess SFT in dogs. The *red* box illustrates the transducer location in each of the five areas used in the study. **b**–**f** Representative RTU images from the five anatomical areas sampled. In each image, measurements of subcutaneous fat thickness (SFT) were taken at three different locations (*white vertical lines*) to estimate a mean SFT value. *M* muscle plan, *B* bladder, *R* ribs’ acoustic shadow

Ultrasound image acquisition was performed from five anatomical sites (Fig. 1), selected according to their suitability from previous research on dogs [15] (flank, abdomen, thigh and lumbar) and also from the authors’ perception of changes in subcutaneous fat deposits in obese dogs (chest). The locations used were:

1. Chest—at the entry of the chest, from the midline to the left, the transducer transversal to the *manubrium sterni*, over the *cleidocephalicus* muscle;
2. Flank—on the dog’s left side, over the ninth intercostal space, just above the costochondral junction, the transducer transversal to the ribs over the *obliquus externus abdominis* muscle;
3. Abdomen—on the left lateral wall of the abdomen, midway on the perpendicular line between the *linea alba* and the tip of the *processus transversus* of the lumbar vertebrae, the transducer in vertical position over the *obliquus externus abdominis* muscle;
4. Thigh—on the inner face of the right thigh, midway on a diagonal line traced from the *tuber ischiaticum* to the *tuberositas tibiae*, the transducer placed transversal to the femur, between the *gracilis* and *semimembranosus* muscles;
5. Lumbar—between the third and the fifth lumbar vertebrae, over the *M. longissimus lumborum* covered by the thoracolumbar fascia, 2–3 cm to the left of the midline, the transducer parallel to the *processus spinosus* of the lumbar vertebrae.

All the RTU images were saved in 640 × 480 JPEG format for subsequent analysis.

### Ultrasound image analysis and subcutaneous fat measurements

To eliminate subjective inter-operator bias, a single experienced operator analysed all the RTU images using ImageJ software (version 1.38x, NIH, USA; http://rsb.info.nih.gov/ij/download.html). Measures of subcutaneous fat thickness were estimated from the thickness of fat deposits located above the muscles displayed in the images, at all five anatomical sites. The skin was included in all thickness measurements. For each image, the average of the measurements taken at the three different locations was considered, so as to eliminate possible fluctuations that might exist in the thickness of subcutaneous fat. The first measurement was taken at the midline of the ultrasound image and the others were obtained equidistantly, 1.5 cm to each side.

### Statistical analyses

Data were analysed using the JMP statistical analysis software (SAS Institute, Cary, NC, USA). A descriptive data analysis for body weight, BCS and subcutaneous fat thickness (SFT) at each point of collection was carried out by mean, standard deviation (SD), range and coefficient of variation (CV). The relationships between the BCS and SFT measurements obtained by RTU image analyses were computed using the correlation analysis procedure. The general linear model (GLM) procedure was used to fit two models showing the associations between BCS and SFT. The first model was developed to evaluate the effect of BCS on SFT at the five anatomical sites considered. In this model, three categories were used, based on BCS [*overweight* (BCS 4), *ideal* (BCS 3) and *lean* (BCS 2)], with the animal’s body size being used as a covariate. The second model included the anatomical region as a factor of influence over SFT. This model used BCS and rump width as co-variables. For both models, least square (LS) means were determined and compared using an F-test protected LSD (least significant difference). A *P* value ≤0.05 was regarded as statistically significant.

## Results

Overall values for body weight, BCS and subcutaneous fat thickness collected from the five selected anatomical sites used in the current study are presented in Table 1.

**Table 1:** Body weight, BCS and subcutaneous fat thickness (SFT) in dogs (n = 28)

Traits	Mean	SD	Minimum	Maximum	CV (%)
Body weight (kg)	17.52	9.71	5.15	33.00	55.41
BCS (scores 1–5)	3.07	0.60	2.00	4.00	19.67
Subcutaneous fat thickness (mm)					
Abdomen	3.15	1.62	1.02	7.88	51.20
Thigh	2.63	1.27	1.11	5.95	48.31
Flank	2.28	0.93	0.98	4.21	40.83
Lumbar	2.89	1.15	1.14	6.31	39.56
Chest	2.39	0.79	1.22	4.02	33.19

Mean, standard deviation (SD), minimum, maximum and coefficient of variation (CV)

Dogs in this study displayed a mean body weight of 17.5 kg, with values ranging from 5 to 33 kg. Median BCS in these 28 dogs was 3, ranging from 2 to 4 points (n = 4, n = 18 and n = 6, for BCS levels 2, 3 and 4, respectively). Animals displayed different body size, namely miniature (n = 4), small (n = 10) and medium-sized dogs (n = 14). Body weight varied noticeably among the dogs, presenting a coefficient of variation of 55.4 %, in contrast with the lower variation observed for BCS (CV = 19.7 %).

Subcutaneous fat thickness varied among different animals and anatomical sites (Table 1). Overall SFT levels obtained with RTU ranged from 1.02 to 7.88 mm with a coefficient of variation between 33.2 and 51.2 %. The highest variations were found in the abdomen (51.2 %), and the lowest in the chest (33.2 %). The coefficient of variation for SFT measurements in the different anatomical sites showed a pattern allowing them to be divided into three groups (Table 1): the abdomen and thigh with the highest variations (CV = 51.2 and 48.3 %, respectively); the flank and lumbar area with intermediate variations (CV = 40.8 and 39.6 %, respectively), and the chest, the area with least variation (CV = 33.2 %). Overall SFT variation was considerably higher than the variation observed for BCS; even when considering only the chest, variation was about 40 % higher in SFT measurements compared to BCS.

Data concerning the correlations between BCS, BW and SFT from the five anatomical sites analysed are presented in Table 2. The correlation between BW and BCS was low (r = 0.230, P > 0.05). In general, the correlations between BW and SFT measurements were also low (r between 0.214, P > 0.05 and 0.598, P < 0.01). The highest correlations between BW and SFT were obtained from the abdomen (r = 0.415, P < 0.05) and thigh (r = 0.598, P < 0.01). However, the correlation between BCS and SFT measurements proved to be high (r between 0.708 and 0.815, P < 0.01). A strong correlation was also obtained between SFT measurements collected from different anatomical sites (r > 0.654, P < 0.01) with emphasis on the correlation between SFT from the abdomen and the lumbar region (r = 0.873, P < 0.01) or the flank (r = 0.847, P < 0.01).

**Table 2:** Correlations between body weight, BCS and subcutaneous fat thickness (in mm) in the five anatomical sites studied (n = 28)

	BCS	Abdomen	Thigh	Flank	Lumbar	Chest
Body weight	0.230 ns	0.415*	0.598**	0.214 ns	0.361 ns	0.268 ns
BCS		0.799**	0.708**	0.815**	0.781**	0.776**
Abdomen			0.807**	0.847**	0.873**	0.799**
Thigh				0.697**	0.744**	0.654**
Flank					0.818**	0.812**
Lumbar						0.839**

Correlation coefficient values connected with ns P > 0.05; * P < 0.05; ** P < 0.01

Subcutaneous fat thickness was significantly affected (P < 0.001) by the anatomical region used to collect RTU images (Table 3). The abdomen displayed the highest SFT (SFT = 3.15 mm), whereas the lumbar and thigh displayed similar SFT (2.89 and 2.63 mm, respectively), and the flank and chest showed the lowest SFT (2.28 and 2.39 mm, respectively; P < 0.05). No differences were found between SFT from lumbar and thigh regions or between SFT from flank and chest. Taking the difference between the largest and the smallest SFT value, a difference of 0.87 mm was observed, corresponding to a 28 % variation.

**Table 3:** Least squares means of subcutaneous fat thickness (in mm) from the five anatomical sites studied

	Subcutaneous fat thickness (mm)
Body region	
Abdomen	3.15^a^
Thigh	2.63^b^
Flank	2.28^c^
Lumbar	2.89^b^
Chest	2.39^c^
Probability	0.001
SEM	0.13

BCS and rump width were used as co-variables

*SEM* standard error of means

^a,b,c^Within a column, least squares means followed by a common letter are not significantly different (P > 0.05)

BCS categories (*lean*, *ideal* and *overweight*) significantly affect SFT measurements (P < 0.001) (Table 4). In general, a decrease in SFT levels at all the five anatomic sites sampled was observed between the *overweight* and *ideal* classes of dogs (P < 0.05) and between the *ideal* and *lean* (P < 0.05) classes. The only exception was the thigh, where measurements remained similar in animals classified as *ideal* and *lean* (P < 0.05). To sum up, differences in SFT measurements between *overweight* and *ideal* animals ranged between 1.21 and 2.63 mm (in the chest and abdomen, respectively), which represented a variation of 48–65 % in SFT levels among these two classes of fatness. Similarly, the variation in SFT between animals classified as *ideal* and *lean* ranged between 0.36 and 1.50 mm (in the thigh and abdomen, respectively), representing a variation of 46–83 % between those two BCS classes. In general, STF values increased between two to fourfold in *overweight* dogs, compared to *lean* dogs.

**Table 4:** Least squares means for subcutaneous fat thickness (in mm) from five anatomical points according to body condition classes

	Abdomen	Thigh	Flank	Lumbar	Chest
Fat classification					
Overweight	5.43^a^	4.54^a^	3.61^a^	4.48^a^	3.45^a^
Ideal	2.80^b^	2.17^b^	2.08^b^	2.65^b^	2.24^b^
Lean	1.30^c^	1.81^b^	1.19^c^	1.61^c^	1.49^c^
Probability	0.001	0.001	0.001	0.001	0.001
SEM	0.38	0.29	0.21	0.28	0.20

Body size was used as co-variable

*SEM* standard error of means

^a,b,c^Least squares means within a column that are followed by a common letter are not significantly different (P > 0.05)

## Discussion

The main purpose of the present study was to establish the association between BCS and subcutaneous fat thickness in five selected anatomic sites in dogs. Previous studies have confirmed the association between BCS and subcutaneous fat thickness estimated from thoracic radiographs [17] or two-dimensional ultrasound images [18], suggesting that both methods would be suitable for use in routine practice, for prediction of body fatness in dogs, besides that they are easily performed at clinics.

The coefficient of variation was higher for BW than BCS. The differences in the size of dogs used in the present study could explain the coefficient of variation observed for BW. As expected the coefficient of variation was lower for BCS since the scores in BCS charts are independent of animals’ weight and essentially refer to the distribution of fatness covering [15]. On this basis, we hypothesised that SFT estimated in RTU images would likewise reflect fat covering and would, therefore, be relatively independent of the size of the dog, though perhaps being dependent on the pattern of fat distribution in a breed. This hypothesis seems to be supported by the weak correlations between BW and BCS observed in the current work, which other studies have also shown [6], as well as those between BW and SFT. These weak correlations could be explained by the size differences among the dogs enrolled in the present study though it cannot be ruled out that breed specificities may also be involved in the distribution of body fat [9]. Dorsteen and Cooper [6] recognised that BCS is not sensitive enough to be used for research, although it may be useful as a tool for routine evaluation of adiposity in management practices.

In this study, the areas used to generate the RTU images included certain areas described as being of predictive value (flank, abdomen, tight and lumbar) [15], as well as what the authors perceived to be the peripheral morphological changes associated with increased overweight status and obesity (chest). Overall, the coefficient of variation differed among the anatomical sites assessed, in particular in the abdominal and thigh areas. These results agree with previous studies on body composition determined by dual-energy x-ray absorptiometry [7] and on subcutaneous fat thickness showing that diverse body regions yield different correlations with BCS [15]. This could result from existing species-specific differences in the pattern of fat deposition among dogs with increased BCS scores. This issue raises an additional concern which should be addressed in future research, namely, to ascertain which anatomical area can best predict fat content across all BCS scores.

The study by Wilkinson and McEwan [15] used A-mode (one-dimensional) ultrasonography, coupled with a high frequency (20 MHz) probe, enhancing the ability to discriminate small variations in SFT in different regions. However, in the present study, the use of a 10 MHz transducer set for small surface areas and in B-mode, was sufficient to detect SFT changes in lean dogs. In their study, Wilkinson and McEwan [15] determined the thickness of subcutaneous fat located between the *panniculus carnosus* (cutaneous muscle) and skin, which was not included in measurements, whereas in the current study SFT measurements included the skin. Also, in that study euthanized animals from a shelter were used, with an increased representation of young animals (below 3 years old), in contrast to our study, which included only clinically healthy privately-owned mature dogs. These major differences could explain the divergence in the results obtained in the present study regarding SFT in the four common anatomical sites assessed, which were particularly notable for maximum and mean SFT values. It is also worth mentioning that mean SFT values obtained in the present study are closer to the values obtained from histological measurements of subcutaneous fatness by Wilkinson and McEwan [15].

Data gathered herein also highlight the need to identify the most suitable anatomical sites to use in RTU prediction of body fat deposits in routine daily practice or research. Seeking the best anatomical site to measure subcutaneous fat has been a concern of other authors [18]. Morooka et al. [18], examining several points along the vertebral column, state that the lumbar region is the most suitable for sampling subcutaneous fat deposits with RTU.

This study revealed that strong associations exist between each of the five anatomical sites assessed; the highest associations were obtained for the abdominal and lumbar regions. Previous studies reported that the thickness of fat deposits in the lumbar area was closely related to the degree of adiposity in dogs [15, 18].

In the present study, dogs were grouped into three main BCS classes; *overweight*, *ideal* and *lean*. According to previous studies, assigning a BCS score may be challenging when animals approach the extremes between two consecutive scores [2, 19]. SFT data gathered in the present study showed that, on average, the difference in fat thickness between *overweight* or *lean* animals compared with *ideal* animals was higher than that reported to occur in body weight when BCS level changes, in accordance with studies using the same scoring scale as the one used here [6]. An overall reduction of 48–65 % was observed in the thickness of subcutaneous fat between *overweight* and *ideal* weighing animals, while the differences between *lean* and *ideal* animals ranged from 46 to 83 %. These results suggest that RTU is sensitive enough to detect smaller variations in subcutaneous fat than those perceived when using BCS in dogs.

All five anatomical regions used to collect RTU images were similarly sensitive to assess differences between categories. Nevertheless, the fat covering was thicker in the abdomen and the lumbar area, and thinner in the flank. Also, these two regions reveal strong correlations with BCS levels, suggesting that they may be the most suitable areas to collect information on SFT. Nevertheless, additional anatomical areas need to be identified, particularly considering that the interest of a site may change according to the pattern of fat deposition throughout different dogs’ developmental stages (young, mature and old). Additionally, to strengthen the results obtained here, further studies should include the extremes of BCS levels.

## Conclusions

In this preliminary study, it was demonstrated that RTU and image analysis can measure subcutaneous fat thickness and detect its changes with higher sensitivity than results obtained with the 5-point scale used for BCS. Additionally, in spite of the high correlations between SFT and BCS in all five anatomic sites analysed, the abdomen and lumbar areas were those where the greatest differences were observed within each category of fatness (*overweight*, *ideal* and *lean*) and will thus be the most useful to distinguish differences in the adiposity of dogs.

## Abbreviations

BCS: body condition scoring
BW: body weight
CV: coefficient of variation
FCI: Fédération Cynologique Internationale
LS: least square
LSD: least significant difference
RTU: real-time ultrasonography
SD: standard deviation
SFT: subcutaneous fat thickness
